# Automating Predictive Phage Therapy Pharmacology

**DOI:** 10.3390/antibiotics12091423

**Published:** 2023-09-08

**Authors:** Stephen T. Abedon

**Affiliations:** Department of Microbiology, The Ohio State University, Mansfield, OH 44906, USA; abedon.1@osu.edu

**Keywords:** active treatment, bacteriophage therapy, biocontrol, biological control, JavaScript, MOI, passive treatment, pharmacodynamics

## Abstract

Viruses that infect as well as often kill bacteria are called bacteriophages, or phages. Because of their ability to act bactericidally, phages increasingly are being employed clinically as antibacterial agents, an infection-fighting strategy that has been in practice now for over one hundred years. As with antibacterial agents generally, the development as well as practice of this phage therapy can be aided via the application of various quantitative frameworks. Therefore, reviewed here are considerations of phage multiplicity of infection, bacterial likelihood of becoming adsorbed as a function of phage titers, bacterial susceptibility to phages also as a function of phage titers, and the use of Poisson distributions to predict phage impacts on bacteria. Considered in addition is the use of simulations that can take into account both phage and bacterial replication. These various approaches can be automated, i.e., by employing a number of online-available apps provided by the author, the use of which this review emphasizes. In short, the practice of phage therapy can be aided by various mathematical approaches whose implementation can be eased via online automation.

## 1. Introduction

Phage therapy is the application of bacterial viruses, more commonly known as bacteriophages or phages, especially toward the control or eradication of bacterial infections such as in animals, including in humans [1,2,3,4,5,6,7,8,9]. This is a subset of the use of phages more generally to control or eradicate nuisance bacteria found in broader environments [10], resulting in so-called phage-mediated biocontrol or biological control of bacteria. More broadly still is the use of viruses as biocontrol agents against organisms other than just bacteria [11]. Key to the successful use of antibacterial, antimicrobial, or biological control agents generally is the attainment of sufficient densities or concentrations of those agents in situ. But what concentrations are sufficient?

Here, I provide means toward answering that question for phages, which to some degree is situation-specific, and particularly so to the extent that one is attempting to minimize or at least reduce the amount of biocontrol agent applied. This involves discussion of a number of mathematical approaches toward gaining an appreciation of the impact of specific phage titers on targeted bacteria. In addition to providing equations that can be readily applied to different phage-treatment scenarios—and which generally are relatively simple, that is, fairly basic in their composition—I provide links to online JavaScript-based calculators which provide numerical solutions (Table 1). Most of these models can be considered to be of phage therapy pharmacodynamics [12], that is, of the anticipated degree of negative impact of a given in situ phage titer on a population of targeted bacteria.

## 2. Predictive Phage Therapy Pharmacology

In this section, I discuss a number of simple mathematical models that collectively can be predictive of the potential for a given phage titer to negatively impact a targeted bacterial population (Section 2.1, Section 2.2, Section 2.3, Section 2.4 and Section 2.5), along with scenarios toward attaining those titers in situ (Section 2.6). Many of these models I have previously discussed, e.g., [12,13,14]. Here, however, the primary aim is one of describing the basis of online calculators which I have developed that implement the various underlying calculations (Table 1).

Abbreviations of terms used in these calculations are summarized in Table 2, and introduced as well throughout the text. It is important to recognize, however, that for clinical or in vivo phage therapy, many of their values can be poorly described in practice, though exceptional can be determinations or at least estimations of initial, in situ phage titers. As a consequence of modeling-input values not necessarily being definite, it can be difficult to match model outputs to therapeutic outcomes. Nevertheless, it can be useful literally to play with models, entering variable and parameters values using the online calculators, as listed in Table 1, to gain a better “feel” for the pharmacodynamics of systems being worked with, that is, especially in terms of the potential for a given in situ phage titer to impact a targeted bacterial population. Alternatively, the presented models may be qualitatively and even quantitatively predictive of in vitro phage therapy experimentation.

Discussed specifically in this section are concepts associated with determining or estimating phage multiplicities of infection (MOIs; Section 2.1), the likelihood of a bacterium being phage adsorbed for a given phage titer (Section 2.2), rates of bacterial declines in number also as functions of phage titers (Section 2.3), how to estimate what phage titers may be required to reduce bacterial numbers to predetermined sufficient levels (Section 2.4), and the use of Poisson distributions in considering the impacts of phage titers on bacterial survival (Section 2.5). This is followed by consideration of in situ phage population growth (Section 2.6).

### 2.1. Multiplicity of Infection

**Figure 1:**
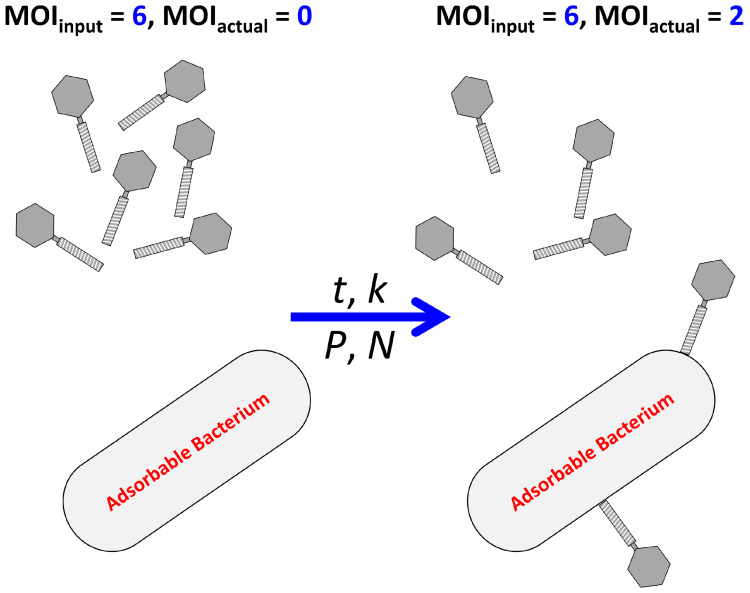
Comparing MOI_input_ with MOI_actual._ On both sides is the same MOI_input_, whereas MOI_actual_ to the right is equal to two vs. equal to zero on the left. Note that, generally, more than one adsorbable bacterium would be present and phages would adsorb over a Poisson distribution (Section 2.5), i.e., with the average number of virions adsorbed per bacterium equal to MOI_actual_. In addition, keep in mind that the quantitative distinction between MOI_input_ and MOI_actual_ results from durations of adsorption periods (*t*) and the phage adsorption rate constant (*k*), the latter defined by a combination of the properties of the adsorbing phages, adsorbable bacteria, and adsorption environment. Phage (*P*) and bacterial (*N*) concentrations, however, also play important roles in determining MOI_actual_, as considered below especially in Equation (5).

Often seen in the phage therapy literature is the concept of multiplicity of infection (MOI). MOIs are relevant due to the statistical nature of phage adsorptions, i.e., such that phage adsorptions [15] are Poissonally distributed across susceptible bacteria [16] (Section 2.5). Though this Poissonal tendency can be quite useful toward appreciating phage therapy pharmacodynamics, the use of MOIs in the phage therapy literature can, in my opinion [12,17], often be problematic. In this section, I consider two different ways of defining phage multiplicities of infection—MOI_input_ vs. MOI_actual_ (Figure 1)—and a way of predicting the latter. An appreciation of these concepts can be useful toward the development of subsequent calculations of phage titer impacts on bacteria.

#### 2.1.1. MOI_input_ vs. MOI_actual_

The two ways of defining MOI are MOI_input_ vs. MOI_actual_ [14,18]. The simplest as well as easiest to use—but the one that is also often misleading [12]—is MOI_input_:

(1) $${MOI}_{input}=P/N,$$

where *P* is a starting phage titer and *N* is the initial concentration of targeted bacteria. This definition, in my opinion, is only useful to phage therapies to the extent that it can be contrasted with determinations as well as predictions of MOI_actual_ (below). Phage therapy dosing based on MOI_input_, in other words, at best should be viewed as “hopeful” since in many cases MOI_input_ does not guarantee nor necessarily even approximate MOI_actual_.

MOI_actual_ instead is the more traditionally used meaning of MOI [19]. It is relevant to phage therapy first because it serves as the basis of Poisson distributions of adsorbed phages over susceptible bacteria and second because the extent of the impact of phages on bacteria also is Poissonal (Section 2.5).

Notwithstanding their distinctions, the definition of MOI_actual_ is similar to that of MOI_input_, though with a clear difference:

(2) $${MOI}_{actual}=P_{adsorbed}/N,$$

with *P*_adsorbed_ not the initial phage titer but instead the concentration, such as per mL, of phages that have adsorbed bacteria, especially as seen after some interval of incubation of free phages with those phage-susceptible bacteria. That is, whereas MOI_input_ is defined in terms of the total number of phages added to bacteria (again, such as per mL), MOI_actual_ is based only on those virions that succeed in adsorbing and, importantly regarding phage therapy, generally only adsorbed phages have an impact on targeted bacteria.

#### 2.1.2. Predicting MOI_actual_

Though not as simple as for MOI_input_, nevertheless MOI_actual_ still can be fairly easy to determine in vitro as

(3) $${MOI}_{actual}=\left(P_{0}-P_{F}\right)/N.$$

Here, *P*_0_ is the starting concentration (titer) of free phages and *P*_F_ is the number free phages remaining unadsorbed following some interval of time (F standing for “Final”), assuming that all free phage losses are due to virion adsorption of targeted bacteria. Unfortunately, determining *P*_F_ can be impractical in vivo. Consequently, it can be helpful instead to be able to predict MOI_actual_. In particular, it can be useful to possess some appreciation of the extent to which targeted bacteria may be impacted by treatment phages, with that impact, for a given phage type, generally being a function of MOI_actual_ (e.g., Section 2.5).

An approximation of the suggested estimation [12,14] can be made based solely on initial phage titers (here shown as just *P*), the phage adsorption rate constant (*k*), and time (*t*):

(4) $${MOI}_{actual}=Pkt.$$

That approximation, however, is useful only at lower bacterial concentrations, e.g., such as below 10^7^/mL, and/or over shorter adsorption intervals, such as over a few minutes rather than over many tens of minutes. In contrast, at all bacterial concentrations or adsorption intervals, one can instead employ

(5) $${MOI}_{actual}=P\left(1-e^{-Nkt}\right)/N.$$

**Figure 2:**
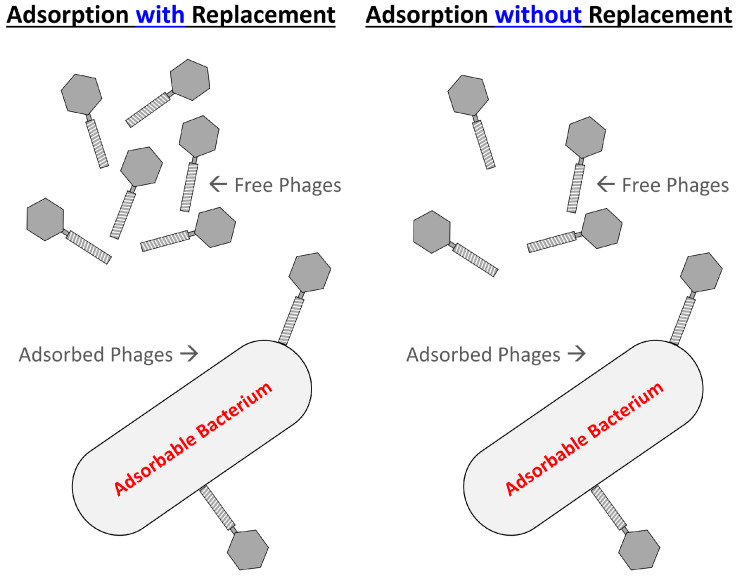
Adsorption with and without replacement of free phages. The mathematically simplified perspective is adsorption with replacement (**left**) since the result is a constant free phage concentration over time. Depending on circumstances, however, that assumption may or may not be realistic. It *may* be realistic, though, if free phage numbers are replaced as a consequence of in situ phage replication or if bacterial numbers are small, thereby resulting in few free phage losses due to adsorption. Alternatively, free phage adsorption without replacement (**right**) explicitly takes into account free phage losses that result from bacterial adsorptions, that is, with free phage concentrations thereby declining over time.

Equation (5) differs from Equation (4) particularly in that it does not assume phage adsorption with replacement; that is, newly adsorbed phages are conceptually replaced with new free phages (Figure 2). Instead, in Equation (5) numbers of free phages are allowed to decline over time as those phages adsorb bacteria, i.e., as is expected in real systems. However, that consideration, as noted, may be qualitatively relevant only when bacterial concentrations are higher or adsorption intervals are longer.

Note in any case that e^−*Nkt*^ goes to zero as *Nkt* becomes larger, i.e., given higher concentrations of targeted bacteria, higher rates of phage adsorption to individual targeted bacteria, and/or longer incubation and thereby longer adsorption times. In that case, to the extent that e^−*Nkt*^ trends toward zero, then MOI_actual_ will in fact come to approximate MOI_input_.

#### 2.1.3. Running the Calculator

The calculation that is presented in Equation (5) is solved via the online multiplicity of infection calculator found at moi.phage.org, there along with solutions to Equations (1) and (4) (with those latter equations solved by the calculator for the sake of comparison). Entering 1 × 10^7^ phages/mL, 5 × 10^6^ bacteria/mL, a 10 min adsorption period, and an adsorption rate constant [12,15] of 2.5 × 10^−9^ mL^−1^ min^−1^ [20] yields an MOI_input_ (as equivalent to MOI_addition_) of 2 but an MOI_actual_ based on Equation (5) instead of 0.25, or 8-fold lower. Additionally, a total of only 1.1 × 10^6^ phages of that original 1 × 10^7^ will be expected to have adsorbed over that interval, while roughly 4 × 10^6^ bacteria/mL will be expected to have remained unadsorbed out of that original 5 × 10^6^, i.e., about 80% of bacteria targeted will not have been phage adsorbed in this example.

### 2.2. Bacterial Likelihood of Being Phage Adsorbed

Related to MOI_actual_, and also solved using moi.phage.org, is simply the likelihood that a targeted bacterium will become phage adsorbed per unit of time, such as per min [15]. An appreciation of this likelihood can be helpful in gaining a better understanding of what may be accomplished upon achieving a given in situ phage titer during treatments. Here, I start with a model of phage adsorption over time and use this to derive the probability of adsorption to a single bacterium over a single unit of time.

#### 2.2.1. Predicting Bacterial Adsorption Likelihood: p(A_c_)

The number of adsorptions predicted to occur per unit time, particularly per unit of volume, such as per mL (*A_t_*), is as follows:

(6) $$A_{t}=NPkt.$$

If we are considering just a single bacterium, then the average number of adsorptions expected (*A*_c_, with the “c” standing for “cell”) can be found simply by setting *N*, the bacterial concentration, to 1 (again keeping in mind that this is all considered as occurring within 1 mL; see Appendix A of [15] for additional detail):

(7) $$A_{c}=1Pkt.$$

This is equivalent to our calculations of MOI_actual_ (Equation (4)). We can then approximate the probability of a single bacterium becoming adsorbed per mL and per min as

(8) $$p\left(A_{c}\right)\approx 1Pk1,$$

and this is particularly so if the number of adsorptions expected per min, *Pk*, is somewhat less than one. If that is not the case, i.e., if the average number of adsorptions per min approaches or exceeds 1, then the probability that a bacterium will become adsorbed by a least one phage over the course of one min can instead be defined as

(9) $$p\left(A_{c}\right)=1-e^{-P\left(1-e^{-Nk}\right)/N},$$

that is, one minus e raised to the opposite of MOI_actual_ as calculated over one min (see Equation (5) for the latter). Note in Equations (8) and (9) that the lowercase “p” stands for “probability” vs. the uppercase, italicized “*P*”, which stands for phage concentration, i.e., phage titer. In those equations, *P* is also implicitly equivalent to *P*_0_ as to is *N* with *N*_0_.

#### 2.2.2. Running the Calculator

The online calculator can be found at adsorptions.phage-therapy.org. By way of example, if we again set *k* to 2.5 × 10^−9^ mL^−1^ min^−1^, for 10^6^ phages/mL (=*P*) the probability that a given bacterium (*N* = 1) will become phage adsorbed over one min, p(*A*_c_), will be 0.0025. For *P* = 10^7^ phages/mL, p(*A*_c_) is instead raised to 0.025. At *P* = 10^8^ phages/mL, the probability is instead 0.25. This is all assuming that phages are adsorbing with replacement, i.e., as specified by Equation (8). If we assume that phages are not adsorbing with replacement, then bacterial concentration (*N*) will come to matter somewhat more. Thus, with *P* = 10^8^ and *N* = 10^7^, the number of adsorptions per bacterium that are expected to occur over one min, which is the exponent in Equation (9), is 0.2469, while for *N* = 10^8^ it is 0.2212, and for *N* = 10^9^, it is 0.0918. These correspond to p(*A*_c_) values of 0.2188, 0.1984, and 0.0877, respectively. The declines seen with greater bacterial numbers in turn are due to substantial losses of free phages to adsorption to the now substantial numbers of bacteria (the *Nk* term in Equation (9)) in combination with there simply being more bacteria for a given number of phages to adsorb (*N* as found in the exponent’s denominator).

These latter calculations come to matter somewhat more if we assume both phage adsorption without replacement and longer adsorption intervals. Thus, for *P* = 10^8^, *N* = 10^8^, and *t* = 60 min, we have an expectation (Equation (5)) of a total (on average) of 1 phage adsorption per bacterium (i.e., in this case 1 = *P*/*N* vs. the 0.2212 indicated in the previous paragraph and p(*A*_c_) = 0.3679). With replacement of free phages following adsorption, however, the expectation (from Equations (4) or (7)) is instead an average of 15 phage adsorptions per bacterium over that same 60 min interval with p(*A*_c_) = 0.0000! Thus, unless phage concentrations can be sustained at high levels—e.g., by adding more phages, targeting smaller numbers of bacteria, or if phages are able sustain their numbers on their own such as due to in situ replication (Section 2.6)—then Equation (7)-type estimations can grossly overestimate expected per-bacterium levels of phage adsorption.

It is important in any case to recognize how dependent these outputs are on the magnitude of *k* [15]. If *k* is smaller, i.e., if we are working with a phage that has a lower potential to adsorb, then p(*A*_c_) too will be smaller. Alternatively, with phages that adsorb faster, the resulting p(*A*_c_) will be larger. These various ideas can be translated directly into what can be described as bacterial half-lives and related decimal reduction times (next section).

### 2.3. Bacterial Reduction Times

One measure of the susceptibility of a microorganism to an antimicrobial agent is what is described as decimal reduction time [14]. This is how long it takes for a given concentration of antimicrobial agent to reduce target numbers by 90% (here, abbreviated as *t*_0.1_). Nearly equivalent mathematically, we can speak of half-lives, which is the time it takes to reduce a target bacterial population by 50% (*t*_0.5_). Alternatively, we can consider reductions by 99% (*t*_0.01_), and so on. In addition, and also similar mathematically, is mean free time (*t*_MFT_), which for our purposes is the amount of time on average that it takes until a given bacterium becomes phage adsorbed. Overall, these constructs, as with likelihoods of bacteria being adsorbed by phages (above), can provide insight into the antibacterial utility of a given in situ phage titer.

Note that generally speaking, *t*_0.5_ < *t*_MFT_ < *t*_0.1_ < *t*_0.01_. This means that half of a bacterial population will become phage adsorbed faster than the average for single bacterium in a population to become phage adsorbed, and in turn it will take even longer for 90% of bacteria to become adsorbed, or indeed for 99% of bacteria to succumb to phage adsorption. In any case, for all of the presented equations in Section 2.3.1 and Section 2.3.2, it is assumed that phages adsorb with replacement, with the without-replacement case addressed instead in Section 2.3.3.

#### 2.3.1. Bacterial Half-Lives: *t*_0.5_, and Also *t*_MFT_

The bacterial mean free time in the presence of phages is simply the inverse of the likelihood of a bacterium being phage adsorbed per unit time, as seen with Equation (8), i.e.,

(10) $$t_{MFT}=1/Pk.$$

The time it takes for one-half of a bacterial population to become phage adsorbed is slightly shorter, owing to the exponential decline associated with phage adsorption, i.e., where more adsorptions in absolute terms by a given population of free phages occur early during adsorption periods rather than later (assuming for that assertion that free phage adsorption is, again, without replacement) while later can be much later. Specifically, we multiply *t*_MFT_ by −ln(0.5) (the 0.5 for half-life), which is equivalent to ln(2). Thus,

(11) $$t_{0.5}=\ln\left(2\right)/Pk=0.69/Pk.$$

#### 2.3.2. Decimal Reduction Times: *t*_0.1_, plus *t*_0.01_

As noted, decimal reduction times simply extend the bacterial reduction to 90% declines, up from the above 50%. The viable (unadsorbed) bacterial population has thus been reduced to 1/10th of its previous size. This can be calculated as

(12) $$t_{0.1}=\ln\left(10\right)/Pk=2.3/Pk.$$

The time it takes to reduce bacterial numbers 100-fold, i.e., to 0.01 of its original number, can be calculated instead as

(13) $$t_{0.01}=\ln\left(100\right)/Pk=4.6/Pk.$$

#### 2.3.3. Phage Adsorption without Replacement

Considering phage adsorption without replacement complicates these formulae somewhat [14], with in the following *x* being equal to the resulting reduction, i.e., such as the above 0.5, 0.1, or 0.01 (and also adding explicitly the zero subscripts for consistency with the following section):

(14) $$t_{x}=t\left(x\right)=-\ln\left(1-ln\left(\frac{1}{x}\right)\frac{N_{0}}{P_{0}}\right)/N_{0}k.$$

Thus, for *x* = 0.5, then 1/*x* = 2; for *x* = 0.1, then 1/*x* = 10, etc. Note, though, that for this equation to be valid then sufficient numbers of phages must be initially present to achieve the indicated reduction, e.g., one must start with *P*_0_ > ln(10)*N*_0_ to achieve decimal reduction or *P*_0_ > ln(2)*N*_0_ phages to reduce unadsorbed bacterial numbers by half.

#### 2.3.4. Running the Calculators

A dedicated, online bacterial half-life calculator can be found at b-half-life.phage.org. Starting with a phage concentration of 10^6^/mL, and an adsorption rate constant as above, then *t*_MFT_ is calculated as 400 min vs. 277 min for *t*_0.5_. Raise the phage titer to 10^7^/mL and these numbers are reduced to 40 and 28 min, respectively, or 4 and 2.8 min given 10^8^ phages/mL (all holding phage titers constant over time). An equivalent calculator, but instead determining phage half-lives as a function of bacterial concentrations, can be found at p-half-life.phage.org. The latter can be used to gain an appreciation of how rapidly a given titer of supplied phages will be expected, as a function of bacterial concentrations (*N*), to become explicitly antibacterial as they adsorb, e.g., such as 50% of those phages adsorbing per min vs. instead 50% per hour. See also Bull and Regoes [21] for an extension of phage half-life calculations to also include phage losses for reasons other than adsorption to phage-infected bacteria.

A decimal reduction, etc., online calculator can be found at decimal.phage-therapy.org. This provides calculations not only for 10- and 100-fold declines in bacterial numbers but also makes this determination both with and without taking starting bacterial concentrations into account. That is, considering phage adsorption both without and with free phage replacement, respectively. The default settings are phage titers of 10^8^/mL and bacterial concentrations of 10^6^/mL. With no decline in phage numbers over time, output is *t*_0.1_ = 9.2 min while *t*_0.01_ = 18.4 min. At such a low bacterial concentration, the equivalent numbers, if assuming instead phage losses to adsorption, are only 9.3 min and 18.9 min, respectively, keeping in mind that total reductions in numbers of unadsorbed bacteria is ten times that for the latter (*t*_0.01_) vs. the former (*t*_0.1_). Raise bacterial concentrations to 10^7^/mL and the equivalent numbers again assuming phage adsorption without replacement instead are 10.5 min and 24.7 min. Then, raise phage titers to 10^9^ (while keeping *N* at 10^7^/mL) and we find that *t*_0.1_ = 0.9 min while *t*_0.01_ = 1.8 or 1.9 min (these latter two values are without losses due to phage adsorption and with losses due to phage adsorption, respectively).

### 2.4. Inundative Phage Quantities

A slightly more sophisticated way of thinking about degrees of phage impact on bacteria is to consider not just durations of treatments in combination with how fast phages are adsorbing, but also how large a reduction in numbers of a bacterial population is desired [12]. This differs from the above bacterial reduction times (Section 2.3) because the sought end points are not fractional declines in bacterial numbers but, instead, are absolute declines. Thus, rather than, for example, a 99% reduction, a reduction to, e.g., 10^3^ bacteria in total is sought. In terms of required starting phage titers, I have dubbed this an “inundative phage density” (IPD_min_), with “density” and “titer” here being used synonymously. Alternatively, there is an “inundative phage number” (IPN_min_), which is the starting absolute number of phages required, that is, rather than starting phage concentrations (the latter again equivalent to “titer” and “density”). As with the other calculations already considered, an implicit assumption is that all targeted bacteria are equally available to phages for adsorption.

In all of these cases, these are minimum values (“min”) because it is assumed that bacterial losses are occurring as calculated whereas less-than-ideal phage adsorption and infection circumstances likely would result in a requirement for more phages than this “min”, such as IPN_actual_ > IPN_min_. Thus, a failure to successfully predict the extent of reductions in bacterial viability in the presence of predicted inundative quantities of phages can be used to indicate the presence of additional phenomena not considered by models. For example, less bacteria killing than expected can be due to not all targeted bacteria being equally available to phages, such as due to the presence of spatial or physiological refuges from phage attack [22]. Lower levels of killing than expected can also be a consequence of outright genetic bacterial resistance to phages and/or instead underestimations of phage adsorption rate constants. Alternatively, greater bacteria killing than expected can be due to the presence of additional antibacterial mechanisms and/or because new phages have been generated in situ (for the latter, see “Active treatment”, below; Section 2.6). In any case, calculations of inundative phage quantities can provide an appreciation of what phage titers should be required to reduce phage-susceptible bacteria to a given total number of remaining bacteria, over a desired length time, particularly as based on the antibacterial action of dosed phages alone.

#### 2.4.1. Inundative Phage Densities: IPD_min_

The minimum titer of phages required to reduce a volume of bacteria to a given amount over a specific span of time, or IPD_min_, can be calculated either assuming or not assuming that these titers remain constant over time (Figure 2). As with the approaches considered above, assuming a constant phage titer simplifies calculations but becomes less valid the higher bacterial concentrations or the longer the time frame over which adsorption is allowed to occur. In any case, both phage and bacterial replication are ignored for these IPD_min_, or IPN_min_, determinations.

The total starting number of bacteria is equal to the volume of the relevant environment (*V*) multiplied by the starting concentration of bacteria (*N*_0_). The final number of bacteria is independent of volume. That is, often when reducing bacterial presence, you want to reduce the number of bacteria to a given lower amount (*N*_F_) rather than to a given lower concentration. Thus, the fraction of bacteria that are expected to survive given the application of some inundative titer of phages will be *N*_F_/*VN*_0_ (total ending bacterial numbers divided by total starting numbers of bacteria) and this fraction, or at least its inverse, is used in the same manner as for, e.g., decimal reduction time calculations (Section 2.3.2). If phage titers can be held more or less constant over time, then the minimum titer of phages required to achieve that fraction of surviving bacteria can be descried as

(15) $${IPD}_{min}=\frac{ln\left(VN_{0}/N_{F}\right)}{kt}.$$

This is the natural log of the fold-decrease in bacterial concentrations, i.e., as equal to 1/*x* in Equation (14), divided by the product of the phage adsorption rate constant and time, with IPD_min_ representing some phage concentration, i.e., *P*. For a 10-fold decline in bacterial numbers—a decimal reduction and thus *x* = 0.1—this would be *P* = ln(10)/*kt*. With rearranging and modifying the abbreviation for time, this is equivalent to the *t*_0.1_ = ln(10)/*Pk*, as seen above in Equation (12). The quantity ln(10) in turn is equal to the MOI_actual_ required to achieve this 10-fold reduction in concentrations of viable bacteria, i.e., 2.3.

Taking into account phage losses due to adsorption to bacteria has the effect of requiring higher starting phage titers, and this can be described instead as

(16) $${IPD}_{min}=\frac{N_{0}\cdot ln\left(VN_{0}/N_{F}\right)}{\left(1-e^{-N_{0}kt}\right)}.$$

This is equivalent to [starting numbers of bacteria] × [MOI_actual_ required to achieve the desired degree of reduction in bacterial numbers] divided by [fraction of added phages which succeed in adsorbing over time, *t*].

#### 2.4.2. Inundative Phage Number: IPN_min_

An alternative perspective is just how many phages are needed to similarly reduce numbers of bacteria as seen for IPD_min_, but without prior knowledge of bacterial concentrations. This approach can be relevant if numbers of bacteria are known or at least can be estimated, but where treatment volumes are less easily determined. There are two ways of going about this. One is to assume that phage titers are known and remain more or less constant or, alternatively, that 100% adsorption of added free phages can be assumed. Missing is the case where phage numbers are instead declining to some intermediate extent, due to phage adsorptions of bacteria, as that extent cannot be calculated without knowledge of bacterial concentrations.

The first case looks simply like

(17) $${IPD}_{min}=\frac{ln\left(N_{T}/N_{F}\right)}{kt},$$

where *N*_T_ is initial, unadsorbed bacterial numbers (“T” standing for “Total”), and this is rather than initial bacterial concentrations. As indicted though, this is again a calculation of IPD_min_, rather than of a minimum inundative phage number (IPN_min_), and this is because required phage titers rather than just phage numbers would be calculated; note also that the numerator again is equivalent to ln(1/*x*). If phage titers are not easily predicted, i.e., as due to phage application to volumes that are not well defined, we need to resort to assuming instead the noted 100% adsorption of added free phages:

(18) $${IPN}_{min}=\frac{N_{T}\cdot ln\left(N_{T}/N_{F}\right)}{1}.$$

IPN_min_ is thus the total number of phages that need to be supplied, but again assuming 100% adsorption. Note that ln(*N*_T_/*N*_F_) is equal to that MOI_actual_ (Section 2.1) required to reduce bacterial numbers from *N*_T_ to *N*_F_, which, in turn, is an *N*_T_/*N*_F_-fold reduction, and equivalently this is (1/*x*)-fold. For example, with a 10-fold reduction, ln(*N*_T_/*N*_F_) = 2.3 = MOI_actual_. IPN_min_, as described by this equation, is therefore equal to that MOI_actual_ multiplied by the total number of bacteria targeted, i.e., by *N*_T_.

If MOI_actual_ should fail to approximate MOI_input_, and the degree of discrepancy is known, then one can modify the previous equation as

(19) $${IPN}_{min}=\frac{N_{T}\cdot ln\left(N_{T}/N_{F}\right)}{{MOI}_{actual}/{MOI}_{input}}$$

We expect, in any case, for MOI_input_/MOI_actual_ ≥ 1 to hold under all circumstances, since it is impossible to adsorb more phages than there are phages (as above, assuming no in situ phage replication). Therefore, the less extensively that phage adsorption occurs, e.g., MOI_input_ ≫ MOI_actual_, even assuming ideal adsorption conditions, then the more phages that will be required to reduce bacterial numbers to an equivalent extent.

#### 2.4.3. Running the Calculator

An online calculator is available for determining inundative phage quantities, as found at inundative.phage-therapy.org. If we start with 10^6^ bacteria/mL (=*N*_0_), and consider only 1 mL of volume (*V*), then reductions to 10^3^ bacteria in total (*N*_F_) over one hour (*t*) requires 4.5 × 10^7^ phages/mL, assuming via Equation (15) that there are no phage losses (=IPD_min_). This changes to 5.0 × 10^7^ phages/mL given phage losses to adsorption, as per Equation (16) (=IPD_min_). Alternatively, via Equation (18), a starting number of only 6.9 × 10^6^ phages (=IPN_min_) is required if 100% phage adsorption is assumed. (Note in the example that 10^6^ is both the starting bacterial concentration and starting bacterial number since only 1 mL is being considered.)

Additional examples of IPD_min_ determinations are found in Table 3, all assuming a value for *k* of 2.5 × 10^−9^ mL^−1^ min^−1^. Notice how nearly the same numbers of phages are required to reduce bacterial numbers to the same amount, e.g., 1 (=10^0^), regardless of starting bacterial numbers. Thus, starting with 10^6^ bacteria/mL in 100 mL requires 1.2 × 10^8^ phages per mL (assuming no phage losses over time) but still half as many phages starting with only 10^2^ bacteria/mL despite the 10,000-fold difference in numbers of starting bacteria. Thus, reducing bacterial populations to a substantial extent requires relatively high phage titers and this is so even if starting bacterial concentrations are relatively low. The explanation for why this is the case has to do with the statistics of Poisson distributions (next section).

### 2.5. Poisson Distributions

A Poisson distribution is similar to a normal distribution except that the *x* axis, defining the independent variable, *r*, consists solely of integers that cannot fall below 0. Thus, *r* = 0, 1, 2, 3, etc. Furthermore, what are varied on the *y* axis are the probabilities associated with each of those integers, i.e., *y* = p(*r*). The magnitude of p(*r*) is defined as follows

(20) $$p\left(r\right)=\frac{n^{r}e^{-n}}{r!}.$$

For our purposes, *r* represents categories of phage adsorptions to bacteria, i.e., the unadsorbed fraction (*r* = 0), the fraction adsorbed by only a single phage (*r* = 1), the fraction adsorbed by two phages (*r* = 2), and so on. In contrast, the variable, *n*, is MOI_actual_ (Section 2.1). Thus, the fraction of bacteria expected within each of the *r* categories is defined for a given MOI_actual_ by a Poisson distribution [16].

#### 2.5.1. Predicting Bacterial Survival

If we set *r* to zero, then Equation (20) is reduced to

(21) $$p\left(0\right)=e^{-n},$$

keeping in mind that 0! = 1, as is also the case for any number raised to zero, i.e., *n*^0^. Rearranging, then *n* = MOI_actual_ = −ln(p(0)) = −ln(*N*_F_/*N*_T_) = −ln(*x*), keeping in mind that ln(1/*x*) = −ln(*x*). *N*_T_ is the starting number of unadsorbed bacteria, i.e., as found prior to phage addition, while *N*_F_ is the ending or “Final” number of unadsorbed bacteria. MOI_actual_ is thus equal to the negative natural log of the fraction of bacteria remaining unadsorbed following some extent of phage exposure, or the positive natural log of the fold-decrease in bacterial numbers.

#### 2.5.2. Killing Titers: *P*_K_

Killing titer (*P*_K_) calculations [12] take the above prediction of bacterial survival and literally rearrange it. This, in contrast to much of the above, is therefore a phage titer determination that is based on bacterial survival rather than a prediction of bacterial survival that is determined, at least in part, by knowledge of initial phage titers. As equivalently seen with Equation (18), MOI_actual_ is multiplied by the initial bacterial concentration, but here with MOI_actual_ calculated based on the fraction of bacteria that have survived, assuming that all added phages have adsorbed:

(22) $$P_{K}=-ln\left(p\left(0\right)\right)N_{0},$$

recalling that MOI_actual_ = −ln(p(0)). Thus, if 10^8^ bacteria per mL are reduced to 10^7^, then the calculated killing titer is −ln(0.1) × 10^8^ = 2.3 × 10^8^. This would be equal to *P*_0_, i.e., the starting phage concentration, assuming that all free phages initially present adsorbed (and that no phage replication has occurred).

Note, though, that the requirement that all free phages must adsorb means that, for this calculation, MOI_actual_ must equal MOI_input_, that is, in order for *P*_K_ to be an actual phage titer determination. If insufficient time is allowed for adsorption, however, then MOI_actual_ will be lower than MOI_input_, resulting in the calculated *P*_K_ being less than *P*_0_. Consequently, killing titer determinations will always underestimate starting phage titers unless complete phage adsorption is allowed to occur, keeping in mind though that often a small fraction of phages will fail to adsorb seemingly no matter what [23,24,25,26]. Of course, for killing titer calculations to hold true, then bacterial replication also must be insubstantial during phage application. Nevertheless, killing titers can provide at least an approximation of what phage titers would have been necessary to achieve the amount of bacteria killing observed, which can in turn be compared with what phage titers actually had been present at the start of phage treatments of a bacterial population.

#### 2.5.3. Running the Calculators

A Poisson frequency calculator is presented at Poisson.phage.org, requiring a single input, that of MOI_actual_. Note that this need not be an integer. For example, for MOI_actual_ = 1.5, the app indicates that the fraction of bacteria expected to not have been phage adsorbed is 0.22 (or 0.37 for MOI_actual_ = 1). Additionally, relevant for certain phage biology experiments is the fraction of bacteria which are singly vs. multiply adsorbed [12]. For MOI_actual_ = 1.5, these fractions are 0.33 and 0.44, respectively, such that, though with rounding error, 1 = 0.22 + 0.33 + 0.44. Also calculated are the fraction of bacteria, of those that have been adsorbed at all, which have been singly vs. multiply adsorbed. For this same example (MOI_actual_ = 1.5), those fractions are 0.43 and 0.57, respectively, which also add up to 1. That is, 43% of bacteria that have been adsorbed in this example are predicted to have been singly adsorbed.

The killing titer calculator can be found at killingtiter.phage-therapy.org. Entered here are concentrations of still-viable bacteria as found both before and after phage adsorption, keeping in mind (again) that for this to be an actual titer determination, then phage adsorption must go effectively to completion, thus requiring sufficient time though also an absence of bacterial replication during that time. If for example you were to start with 10^7^ bacteria/mL and end up with 10^4^ bacteria/mL, then your calculated killing titers would be 6.9 × 10^7^ phages/mL. Additionally, MOI_actual_ is calculated, which in this example, would be 6.9. The greatest utility of such killing titer determinations is for use toward establishing the titers of phages—or other agents such as phage tail-like bacteriocins—which, for whatever reason, are unable to form plaques on the bacterial strain being targeted, while still possessing single-hit kinetics of those bacteria [21]. Nevertheless, it is also useful to compare calculated killing titers (*P*_K_) with actual titers (*P*_0_) to assess treatments, with *P*_K_ < *P*_0_ implying a less-than-ideal phage impact while *P*_K_ > *P*_0_ would imply instead a greater-than-expected phage impact.

### 2.6. Active Treatments

All of the above-discussed approaches ignore both phage and bacterial population growth. In my opinion, ignoring bacterial population growth is reasonably justified, particularly (1) if one is treating bacterial populations which already are somewhat mature in terms of not displaying substantial additional bacterial population growth or (2) if phage impact is fast relative to rates of bacterial growth. The latter generally can be achieved by supplying phages in high concentrations, i.e., inundative densities acting over relatively short periods.

Phage in situ replication, on the other hand, is less easily ignored, except in a limited number of circumstances, e.g., such as application of overwhelming phage numbers, so-called passive treatments [27,28,29,30], or when phages are used which are unable to replicate [21,31]. Such phage replication, giving rise to in situ phage population growth during phage therapies, can result in what have been described as active treatments [27,28,29,30], with “Active” referring to a relevance of virion-productive phage infections of bacteria, again in situ, toward enhancing phage therapeutic efficacy. Importantly, however, we can also differentiate phage in situ population growth into that associated with high vs. low overall bacterial concentrations and also that phage population growth occurring in association with vs. without bacterial clumping or clustering, that is clumping or clustering of bacteria such as into biofilm microcolonies. Thus, for example:

1. Low bacterial concentrations *without* clumping and lower starting phage titers. In the case of low bacterial concentrations and no bacterial clumping, phage population growth likely is mostly irrelevant, since in situ phage replication will not be expected to have a substantial impact on more “global” phage titers. That is, bacteria are present in insufficient quantities to produce relatively large concentrations of new phages across environments. Still, these circumstances, given sufficient environmental mixing, are easily modelled mathematically.
2. Low bacterial concentrations *with* clumping and lower starting phage titers. With spatial structure in combination with bacteria being found in clonal clusters—but bacteria nonetheless overall found at low concentrations—phage in situ replication could in fact be relevant, though not globally, and the mathematics portraying such situations is not straightforward. I describe this latter scenario as a *locally* active treatment [32].

In other words, for the latter, once a bacterial microcolony has been infected by a single phage, it is not unlikely that other bacteria found in the same microcolony or cellular arrangement will be impacted by resulting locally produced phage progeny [33,34]. It is just that those newly generated phages, if amplified in number from only sparsely available bacterial microcolonies, may be unlikely to easily find other bacterial microcolonies to infect, due to those phages not achieving relatively high titers across treated environments.

Higher bacterial concentrations with bacterial clumping, such as existing as biofilms, again greatly complicate the necessary mathematics and are therefore not straightforward to model. Without clumping, though, we still may describe two basic scenarios when considering the treatment of higher bacterial concentrations, distinguishing passive from active treatments. These are:

3. Higher bacterial concentrations without clumping and *higher* starting phage titers. First is the noted passive treatment in which phage in situ replication is not required to achieve desired levels of bacterial eradication, e.g., as due to the employment of inundative phage concentrations (Section 2.4). This is because sufficient quantities of phages have been supplied via phage dosing alone.
4. Higher bacterial concentrations without clumping and *lower* starting phage titers. Second is what I have described as *globally* active treatment [32]. Here, the assumption is that phage virions are free to diffuse relatively rapidly about environments or otherwise be readily moved about, such as within blood. Therefore, phages produced in one location can give rise to sufficient increases in phage titers, i.e., to inundative densities (Section 2.4.1) throughout a phage-treated environment.

It is this globally active treatment that is the primary focus of this section and indeed, this is how active treatments typically are envisioned, at least from modeling perspectives [27,28,29].

As follows, I first take into account phage population growth (Section 2.6.1) and then bacterial population growth (Section 2.6.2), both in terms of such globally active treatment. In Section 2.6.3, the two approaches are brought together, with an online calculator for running the resulting model introduced. Section 2.6.4 then extends the idea of modeling phage active treatments but reviewing in vitro experimentation in particular, rather than in silico modeling.

#### 2.6.1. Considering Phage Population Growth

Modeling of phage population growth as well as bacterial population growth has typically been performed within a context of chemostat-based phage–bacteria community dynamics, e.g., [35,36]. Here, for simplicity as well as because it likely is at least equivalently relevant to phage therapy, only batch-culture-type scenarios are considered, e.g., as modeled in Abedon et al. [37]. Batch- vs. chemostat-based modeling is equivalent, except that inflow of new media and outflow of culture media along with bacteria and phages is not considered with batch growth. Additionally, here no bacterial-concentration-associated constraints on phage or bacterial growth rates are considered [36,38].

Though models of phage population growth are often presented based on calculus (as reviewed in Stopar and Abedon [13]), in reality their numerical solutions will typically employ discrete iterations, e.g., advancing simulations in one-minute intervals. Therefore, the relevant equations are presented here explicitly as these iterated equations. Thus:

(23) $$P_{t+1}=P_{t}+BkP_{t-L}N_{t-L}e^{-LI_{N}}-kP_{t}N_{t}-I_{P}P_{t}.$$

This can be expressed in words as follows: The phage concentration found one interval later (*P_t_*_+1_) is equal to the just-previous phage concentration (*P_t_*) plus new phages generated upon phage-induced bacterial lysis (*B* meaning burst size) of those bacteria infected one latent period (*L*) earlier (*BkP_t_*_−*L*_*N_t_*_−*L*_). Subtracted from this are those phages lost to adsorption (*kP_t_N_t_*) along with any free phages lost for any additional reasons (*I_P_P_t_*). In addition is the construct, $e^{-LI_{N}}$, which has the effect of removing phage-infected bacteria that have been lost to non-phage-related decay over the course of one latent period. *I_N_* is defined as the rate of loss of bacterial cells for non-phage-related reasons, as is also employed in the following section.

#### 2.6.2. Considering Bacterial Population Growth

Bacterial growth is introduced as an additional iterated equation, one which feeds into the equation modeling phage population growth (Equation (23)) and vice versa. The growth itself is modeled using what is known as the Malthusian parameter (*μ*), which basically is the rate of exponential growth of the population as occurs over a single interval, such as one minute. Thus,

(24) $$N_{t+1}=N_{t}+\mu N_{t}-kP_{t}N_{t}-I_{N}N_{t}$$

This—similar to the phage equation, Equation (23)—is the concentration of unadsorbed bacteria one interval later (*N_t_*_+1_) as equal to the just-previous unadsorbed bacterial concentration (*N_t_*) but also with new bacteria being added due to bacterial binary fission (*μN_t_*). Bacteria are lost to phage adsorption (*kP_t_N_t_*) as well as to phage-unrelated forms of inactivation (*I_N_N_t_*). As with modeling phage population dynamics, inactivation can be ignored (that is, by setting the parameter, *I_N_*, to zero). Alternatively, by setting *I_N_* and *I_P_* to the same value, then a chemostat-like system can be modeled, with both parameters thereby describing outflow. Inflow in any case can be ignored because, as noted, nutrient concentrations are not being considered.

#### 2.6.3. Running the Calculator

Running a simulation based on the above two equations involves simply employing appropriate parameter values along with starting conditions and then stepping through both equations one interval at a time. There is increasing imprecision the longer the incrementation interval, but I have found that resulting error is minor given use of one min intervals. I have had a tendency to employ a spreadsheet, such as Microsoft Excel^®^, to run these sorts of simulations [13], which involves stepping through the equations vertically in columns, with each row corresponding to one interval. Alternatively, an online calculator for modeling globally active treatment can be found at active.phage-therapy.org, though there this is described simply as “Active treatment”.

The default, though otherwise fully adjustable parameters entered in the online calculator are latent period (15 min), burst size (100), initial phage titer (10^7^/mL), a phage inactivate rate (0.00001 as a per min fractional loss), an initial bacterial concentration (10^3^/mL), the Malthusian parameter (0.013), a phage-independent rate of bacterial loss (also set to 0.00001 and which, as also for phages, is set there deliberately small by default), and a simulation duration (60 min). Running the calculator using those inputs yields an only minor, log_10_ 0.003 increase in phage titers, owing to the very small starting bacterial concentration. This is roughly a 1% increase in phage numbers. In contrast, bacterial concentrations over this span are reduced by log_10_ 0.317, which corresponds to a 52% reduction. Change the starting bacterial concentration to 10^6^/mL and over that hour, phage concentrations increase by 1300% (1.146 log_10_, which is from 10^7^ to ~1.4 × 10^8^ phages/mL) while bacterial concentrations decline by 3.834 logs (down to 1.5 × 10^2^/mL, or nearly a 100% decline). Thus, in this latter case, there are sufficient bacteria present to support substantial phage population growth, and this in turn results in more substantial declines in numbers of bacteria, i.e., considerably effective active treatment is occurring. The caveats, however, are that it is difficult to determine in situ phage latent periods or burst sizes as well as bacterial rates of replication, and indeed, determining in situ phage adsorption rate constants as well. Furthermore, it is difficult to assume that in situ environments are homogeneous, or necessarily well mixed, both as required implicitly by the simulation. Still, this calculator allows one to easily play a number “what if?” scenarios regarding starting phage and bacterial concentrations.

#### 2.6.4. Additional Approaches to Predicting In Situ Efficacy, from In Vitro Characteristics

A major issue with such mathematical modeling of active treatments, toward prediction of in situ phage behavior as presented above, is that it is labor intensive to obtain the needed parameter values, even in vitro, especially those of phage latent periods, phage burst sizes, phage adsorption rate constants, and bacterial growth rates. Consequently, efforts have been made to model active treatments experimentally, also in vitro, again toward predicting phage abilities during actual treatments, but without going through the struggle of obtaining those individual measures. These efforts, such as found in [39,40,41], particularly involve determinations of phage impacts on in vitro optical densities (i.e., turbidity) of broth bacterial cultures over time, as summarized in terms of areas under the curve (AUCs); see also [42,43] for review of this general broth-culture approach. Smaller AUCs (less overall bacterial culture density over time) are indicative of higher phage antibacterial virulence—due to sooner, faster, or more complete phage-induced lysis of bacterial populations—and larger AUCs indicate lower phage antibacterial virulence.

An issue with these optical density-based approaches is, unfortunately, the occurrence of lysis inhibition [44], which has the effect of retaining and even boosting the turbidity of bacterial cultures despite the productive lytic phage infection of most or all phage-sensitive bacteria present. Importantly, though, only a subset of phage types display this phenotype. Nevertheless, see panel B in Figure 1 of [39], where the phage T4 studied there is historically well known for displaying the lysis inhibition phenotype [45,46,47], and see also, e.g., [48,49,50] for examples of lysis inhibition as displayed by other phages. This issue of lysis inhibition was a constraint also on our own work, studying phage broth performance using an optical density approach, which limited what phages we were able to analyze [51].

Optical-density-based in vitro modeling approaches, to a degree, build on earlier work where in vitro determined phage population growth rates, presumably a key measure of active treatment effectiveness, were found to correlate with in vivo phage therapy efficacy [52,53]. Those efforts involve measuring increases in phage prevalence over time, an approach that should in fact mostly not be impacted by lysis inhibition, and this is instead of the above-noted optical-density measures of decreases in bacterial prevalence. To a degree, though, contrast those correlations between in vitro phage growth rates and in vivo efficacy with the in vivo observations of Bull et al. [54], which clearly indicate that in vivo phage population growth is not necessarily always a robust predictor of phage antibacterial effectiveness. Indeed, phage population growth rates presumably are far more relevant toward active treatment efficacy than toward instead passive treatment efficacy, since the latter, by definition, does not require phage population growth (i.e., as discussed at the start of Section 2.6). Nonetheless, determining rates of phage population growth is simpler to accomplish than determining separately phage latent periods, burst sizes, and adsorption rates. (For protocols determining the latter, see [55] along with adsorption rate-determination citations found in [15].) Bacterial turbidity measurements are, in turn, somewhat less labor intensive to obtain than phage population growth rates, especially given the use of kinetic microplate readers vs. plaque-based measures of changes in phage titers over time.

A limitation for all of these approaches, including the mathematical modeling emphasized here, is that resulting predictions of subsequent phage performance during therapies will only be as useful as experimental in vitro environments are representative of subsequent in situ conditions [56,57,58]. Nonetheless, the goal with all of these methods is to gain a more robust appreciation of what at least might be achievable by a given phage during an actual treatment rather than choosing phages for phage therapy based solely on more simplistic measures of host range such as just spotting or just plaquing abilities [43,59,60], or instead relying upon genome sequence-based methods for phage host-range determination, the latter as currently are under development [61,62].

In any case, when models of phage treatments—whether in vitro, in silico, or indeed in vivo—indicate a lower likelihood of phage therapy success, then that should bode less well, ultimately, for treatment effectiveness than if these models instead suggest a higher potential for attainment of antibacterial efficacy.

## 3. Discussion

The strengths of the various approaches provided here stem not just from their simplicity but also from their bases in mechanistic modeling. Specifically, a phage life cycle consists of virion infection and release which is then followed by virion movement and then adsorption. All of these processes are well studied, including at the whole-organism levels that are considered here. That is, phage populations adsorb following well-studied models of exponential decline in free phage numbers, models which were first published on in the early 1930s [63,64]. The duration of phage infections and resulting burst sizes were first studied quantitatively later in that same decade, now over 80 years ago [65], and the use of Poisson distributions to describe phage adsorptions has been around for almost as long [16]. Models of the type, as utilized here under the heading of “Active treatments” (Section 2.6), can be traced at least to Campbell in 1961 [38] and have been exploited extensively by Levin and colleagues [66,67,68,69], the latter starting in 1977 [35] (see also, e.g., [21,27,28,56,70,71,72,73,74,75,76,77,78,79,80,81,82,83]). An important goal of those studies is the use of models and parameter values that allow some predictive power despite the complexity of the phage–bacteria community dynamics that these studies have sought to emulate.

I have been especially involved in analysis of the earliest work of Bohannon and Lenski [36]. They used *Escherichia coli* B and bacteriophage T4 in a minimal-medium chemostat experiment, which they followed for 200 h prior to the takeover of their cultures by phage-resistant bacteria. Notably, their model provided predictions that were more qualitative than quantitative, that is, consistent with trends but less consistent with actual phage titers and bacterial concentrations, even prior to phage-resistant bacteria coming to dominate populations. Particularly since the phage they employed (T4) displays the complicating phenotype of lysis inhibition [44] (see also Section 2.6.4), I sought to improve the quantitative predictive power of their model [84]. The result of a number of modifications (Appendix A) was a substantial increase in predictive power through the first 100 h of their actual chemostat. (See also Abedon et al. [37], their Figure 2, for explicit validation of the ability of models such as those presented here to predict phage population growth rates again in vitro. See also Figure 1 of Weld et al. [71].)

These comparisons between experiments and models, in combination with the long history of study of these sorts of mechanistic phage–bacteria community dynamics modeling, or simply of phage population dynamics, suggests that though the approaches provided in this article may not be 100% predictive, they are likely as close in their predictive power as the precision that phage therapy experiments themselves will tend to be monitored. This, though, comes with the caveat that modeling outputs are only as good as modeling inputs, meaning that knowledge of actual phage adsorption rates as well as latent periods and burst sizes for active treatments can be relevant to predicting phage therapy outcomes. Alternatively, failures of models to accurately predict outcomes can be suggestive of a less-than-ideal appreciation of the magnitudes of those phage growth parameters in situ.

## 4. Conclusions

Though I suggest that modeling can have a place in gaining a better understanding of the pharmacology of phage therapy, more sophisticated phage therapy models [77,79,82,83] may be less accessible to the typical phage therapy practitioner or less useful in terms of application to novel circumstances. Alternatively, and at the other extreme, dismissing mathematical descriptions of phage treatments altogether seems as though it can, if my reading of the phage therapy literature is any indication, result in reduced understanding of phage treatments and their outcomes than should otherwise be possible. Explicitly, I typically employ simple mathematical constructs to better understand the underlying pharmacology, particularly pharmacodynamics, of published phage-bacterial interactions, e.g., [85]. It is my feeling that such applications might be as useful *prior to* the publication of phage therapy studies as they can be to me when analyzing studies *following* their publication, hence the emphasis of this review.

A different consideration is the utility of these various mathematical approaches to clinical phage therapy. My suspicion, in fact, is that in explicit terms, this math may be less useful than can be the case for preclinical studies, if only because there is less opportunity to make the detailed measurements that many of these models require, e.g., such as of bacterial concentrations, phage titers in association with targeted bacteria, phage adsorption rate constants, phage burst sizes, etc. These are all as found in situ while treating infections caused by what are typically somewhat uncharacterized bacterial strains and, in many cases, also in combination with antibiotics [41,57,86,87,88,89], which can have antagonistic impacts on phage infection abilities [41,51,85,90]. In particular for the latter, note that of 18 clinical phage therapy studies that I was able to obtain—published in 2023 or, at the time of writing, which are published but still online ahead of print—at least 16 indicate treatments using phages in combination with antibiotics [57,91,92,93,94,95,96,97,98,99,100,101,102,103,104,105,106,107,108]. See also [109], where 79 of the 114 clinical phage treatments reported “were administered in combination with standard-of-care antibiotics”.

Notwithstanding the greater modeling imprecision which inevitably results when transitioning from pre-clinical studies to real-world phage therapy implementation, it is unlikely to be productive for clinical phage therapists to be unaware of the various presented models and especially their outputs. Thus, my suggestion, effectively for all phage therapists—whether or not they choose to explicitly apply these methods to specific phage therapies—is nonetheless to “play” with these models. That is, to run the described online calculators (Table 1) using different input values, e.g., by varying in situ phage titers or targeted bacterial concentrations, and to do so simply to gain an appreciation for how changing treatment approaches or conditions might impact treatment effectiveness. The goal should be to gain greater understanding especially of what phage doses can be more or less likely to result in sought phage treatment efficacies.

## Figures and Tables

**Table 1:** Summary of web pages referred to and their URLs (Uniform Resource Locators).

Topic	Section	URL
Multiplicity of Infection	Section 2.1	moi.phage.org
Phage Adsorptions	Section 2.2	adsorptions.phage-therapy.org
Bacterial Half-Life	Section 2.3.1	b-half-life.phage.org
Decimal Reduction Time	Section 2.3.2	decimal.phage-therapy.org
Phage Half-Life	Section 2.3.4	p-half-life.phage.org
Inundative Phage Quantities	Section 2.4	inundative.phage-therapy.org
Poisson Frequencies	Section 2.5	Poisson.phage.org
Killing Titers	Section 2.5.2	killingtiter.phage-therapy.org
Active Phage Therapy	Section 2.6	active.phage-therapy.org

**Table 2:** Relevant Parameters and Variables.

Abbreviation	Description	Comments
*A*_c_	Bacterial probability of being adsorbed	Likelihood of an individual bacterial cell being adsorbed per unit time, e.g., 1 min; the “c” stands for “cell”
*A_t_*	Adsorptions over time	Number of phage adsorptions that occur over some interval of time, *t*
*B*	Burst size	Number of virions produced per phage infection; might range from 10 to well in excess of 100
e	Base of the natural logarithm	=2.718… (a non-repeating decimal)
*I*_P_, *I*_N_	Decay rate	Rates of loss of free phages (*I*_P_) or bacteria (*I*_N_) that occur for reasons that are independent of phage adsorption
IPD_min_	Inundative phage density	Minimum phage titer required to reduce a bacterial population from some starting number to some ending number over some specified interval of time, not assuming 100% phage adsorption
IPN_min_	Inundative phage number	Minimum phage titer to achieve the same as IPD_min_ except here assuming 100% phage adsorption
*k*	Adsorption rate constant	Probability that one virion will adsorb one bacterium as suspended in a unit volume of fluid (e.g., 1 mL) over the course of some unit time (e.g., 1 min), hence, e.g., mL^−1^ min^−1^ units, though often expressed instead as mL min^−1^
*L*	Latent period	Measure of the length of infection by a phage a bacterium
ln	Natural logarithm	For example, ln(2) = 0.69 = −ln(0.5) = −ln(1/2); ln(e) = 1
MOI_actual_, *n*	Actual multiplicity of infection	Number of adsorbed phages divided by the number of adsorbable bacteria; equivalent to *n* as used in Poisson calculations
MOI_input_ or MOI_addition_	Input multiplicity of infection	Number of phages added to targeted bacteria divided by the number of those bacteria
*Μ*	Malthusian parameter	A measure of bacterial population growth rate in per time units
*N*, *N*_0_, *N_t_*	Bacterial concentrations	Subscript 0 refers to initial concentrations, though in many cases this is implied so the subscript is not always present; subscript *t* refers to the concentration of unadsorbed bacteria following a previous time interval, *t*
*N*_F_, *N*_T_	Bacterial numbers	Subscript F refers to a “Final” number of unadsorbed bacteria; subscript T refers to “Total” and is used instead of *N*_0_ to distinguish starting bacterial concentration (*N*_0_) from starting bacterial numbers (*N*_T_)
p	Probability	This is lower-case “p” without italicization
*P*, *P*_0_, *P*_F_, *P_t_*	Phage titer	Subscripts are equivalent to those of *N*_0_, *N*_F_, *N_t_*, with *P* in all cases referring to phage concentrations, i.e., phage titers
*P*_adsorbed_	Prior titer of adsorbed virions	Number of previously free phages that have now adsorbed, divided by volume, as to be distinguished from *P*_0_
*P*_K_	Killing titer	Titer of phages required to reduce a bacterial population from a given starting number to a given ending number, assuming 100% adsorption
*r*	Poisson category	Here, e.g., 0 phages adsorbed, 1 phage adsorbed, etc., all per bacterium
*r*!	*r* factorial	For example, 3! = 1 × 2 × 3; 2! = 1 × 2; 1! = 1; 0! = 1
*t*	Time	Generally, here, this is an interval over which adsorption occurs
*t*_0.1_, *t*_0.01_	Decimal reduction time(s)	Time it takes for 90% of unadsorbed bacteria to become adsorbed (*t*_0.1_) or 99% (*t*_0.01_)
*t*_0.5_	Bacterial half-life	Time it takes for one-half of unadsorbed bacteria to become adsorbed
*t*_MFT_	Mean free time	Average length of time it takes for a bacterium to become phage-adsorbed
*V*	Volume	Volume that targeted bacteria and targeting phages are suspended in during phage treatments
*x*	Fraction bacteria	As surviving following phage exposure (=*N*_F_/*N*_T_)

**Table 3:** Calculating inundative phage quantities for one-hour treatments *.

*N*_T_ →	10^10^	10^9^	10^8^	10^7^	10^6^	10^5^	10^4^	10^3^	10^2^	
*VN*_T_ →	10^12^	10^11^	10^10^	10^9^	10^8^	10^7^	10^6^	10^5^	10^4^	
*N*_F_ ↓										
10^−3^	2.3 × 10^8^	2.1 × 10^8^	2.0 × 10^8^	1.8 × 10^8^	1.7 × 10^8^	1.5 × 10^8^	1.4 × 10^8^	1.2 × 10^8^	1.1 × 10^8^	Eq. (15)
10^−3^	3.5 × 10^11^	3.2 × 10^10^	3.0 × 10^9^	3.6 × 10^8^	1.8 × 10^8^	1.5 × 10^8^	1.4 × 10^8^	1.2 × 10^8^	1.1 × 10^8^	Eq. (16)
10^−3^	3.0 × 10^11^	2.8 × 10^10^	2.5 × 10^9^	2.3 × 10^8^	2.1 × 10^7^	1.8 × 10^6^	1.6 × 10^5^	1.4 × 10^4^	1.2 × 10^3^	Eq. (18)
										
10^−2^	2.1 × 10^8^	2.0 × 10^8^	1.8 × 10^8^	1.7 × 10^8^	1.5 × 10^8^	1.4 × 10^8^	1.2 × 10^8^	1.1 × 10^8^	9.2 × 10^7^	Eq. (15)
10^−2^	3.2 × 10^11^	3.0 × 10^10^	2.8 × 10^9^	3.3 × 10^8^	1.7 × 10^8^	1.4 × 10^8^	1.2 × 10^8^	1.1 × 10^8^	9.2 × 10^7^	Eq. (16)
10^−2^	2.8 × 10^11^	2.5 × 10^10^	2.3 × 10^9^	2.1 × 10^8^	1.8 × 10^7^	1.6 × 10^6^	1.4 × 10^5^	1.2 × 10^4^	9.2 × 10^2^	Eq. (18)
										
10^−1^	2.0 × 10^8^	1.8 × 10^8^	1.7 × 10^8^	1.5 × 10^8^	1.4 × 10^8^	1.2 × 10^8^	1.1 × 10^8^	9.2 × 10^7^	7.7 × 10^7^	Eq. (15)
10^−1^	3.0 × 10^11^	2.8 × 10^10^	2.5 × 10^9^	3.0 × 10^8^	1.5 × 10^8^	1.2 × 10^8^	1.1 × 10^8^	9.2 × 10^7^	7.7 × 10^7^	Eq. (16)
10^−1^	2.5 × 10^11^	2.3 × 10^10^	2.1 × 10^9^	1.8 × 10^8^	1.6 × 10^7^	1.4 × 10^6^	1.2 × 10^5^	9.2 × 10^3^	6.9 × 10^2^	Eq. (18)
										
10^0^	1.8 × 10^8^	1.7 × 10^8^	1.5 × 10^8^	1.4 × 10^8^	1.2 × 10^8^	1.1 × 10^8^	9.2 × 10^7^	7.7 × 10^7^	6.1 × 10^7^	Eq. (15)
10^0^	2.8 × 10^11^	2.5 × 10^10^	2.3 × 10^9^	2.7 × 10^8^	1.3 × 10^8^	1.1 × 10^8^	9.2 × 10^7^	7.7 × 10^7^	6.1 × 10^7^	Eq. (16)
10^0^	2.3 × 10^11^	2.1 × 10^10^	1.8 × 10^9^	1.6 × 10^8^	1.4 × 10^7^	1.2 × 10^6^	9.2 × 10^4^	6.9 × 10^3^	4.6 × 10^2^	Eq. (18)
										
10^1^	1.7 × 10^8^	1.5 × 10^8^	1.4 × 10^8^	1.2 × 10^8^	1.1 × 10^8^	9.2 × 10^7^	7.7 × 10^7^	6.1 × 10^7^	4.6 × 10^7^	Eq. (15)
10^1^	2.5 × 10^11^	2.3 × 10^10^	2.1 × 10^9^	2.4 × 10^8^	1.2 × 10^8^	9.3 × 10^7^	7.7 × 10^7^	6.1 × 10^7^	4.6 × 10^7^	Eq. (16)
10^1^	2.1 × 10^11^	1.8 × 10^10^	1.6 × 10^9^	1.4 × 10^8^	1.2 × 10^7^	9.2 × 10^5^	6.9 × 10^4^	4.6 × 10^3^	2.3 × 10^2^	Eq. (18)
										
10^2^	1.5 × 10^8^	1.4 × 10^8^	1.2 × 10^8^	1.1 × 10^8^	9.2 × 10^7^	7.7 × 10^7^	6.1 × 10^7^	4.6 × 10^7^	3.1 × 10^7^	Eq. (15)
10^2^	2.3 × 10^11^	2.1 × 10^10^	1.8 × 10^9^	2.1 × 10^8^	9.9 × 10^7^	7.7 × 10^7^	6.1 × 10^7^	4.6 × 10^7^	3.1 × 10^7^	Eq. (16)
10^2^	1.8 × 10^11^	1.6 × 10^10^	1.4 × 10^9^	1.2 × 10^8^	9.2 × 10^6^	6.9 × 10^5^	4.6 × 10^4^	2.3 × 10^3^		Eq. (18)
										
10^3^	1.4 × 10^8^	1.2 × 10^8^	1.1 × 10^8^	9.2 × 10^7^	7.7 × 10^7^	6.1 × 10^7^	4.6 × 10^7^	3.1 × 10^7^	1.5 × 10^7^	Eq. (15)
10^3^	2.1 × 10^11^	1.8 × 10^10^	1.6 × 10^9^	1.8 × 10^8^	8.3 × 10^7^	6.2 × 10^7^	4.6 × 10^7^	3.1 × 10^7^	1.5 × 10^7^	Eq. (16)
10^3^	1.6 × 10^11^	1.4 × 10^10^	1.2 × 10^9^	9.2 × 10^7^	6.9 × 10^6^	4.6 × 10^5^	2.3 × 10^4^			Eq. (18)
										
10^4^	1.2 × 10^8^	1.1 × 10^8^	9.2 × 10^7^	7.7 × 10^7^	6.1 × 10^7^	4.6 × 10^7^	3.1 × 10^7^	1.5 × 10^7^		Eq. (15)
10^4^	1.8 × 10^11^	1.6 × 10^10^	1.4 × 10^9^	1.5 × 10^8^	6.6 × 10^7^	4.6 × 10^7^	3.1 × 10^7^	1.5 × 10^7^		Eq. (16)
10^4^	1.4 × 10^11^	1.2 × 10^10^	9.2 × 10^8^	6.9 × 10^7^	4.6 × 10^6^	2.3 × 10^5^				Eq. (18)
										
10^5^	1.1 × 10^8^	9.2 × 10^7^	7.7 × 10^7^	6.1 × 10^7^	4.6 × 10^7^	3.1 × 10^7^	1.5 × 10^7^			Eq. (15)
10^5^	1.6 × 10^11^	1.4 × 10^10^	1.2 × 10^9^	1.2 × 10^8^	5.0 × 10^7^	3.1 × 10^7^	1.5 × 10^7^			Eq. (16)
10^5^	1.2 × 10^11^	9.2 × 10^9^	6.9 × 10^8^	4.6 × 10^7^	2.3 × 10^6^					Eq. (18)

* Arrows are used to indicate what values the upper-left abbreviations are describing. *N*_T_ refers to starting bacterial numbers within 1 mL, *VN*_T_ refers to starting bacterial numbers here within 100 mL, and *N*_F_ refers to ending bacterial numbers, with the value N_T_ in its two instances being used equivalent to N_0_. Stacked quantities from top to bottom are IPD_min_ assuming constant phage titers over time (Equation (15)), IPD_min_ not assuming constant phage titers over time (Equation (16)), and IPN_min_ (Equation (18)). “Equation” in the last column has been abbreviated as “Eq.”

## Data Availability

Not applicable.

## Appendix A. Improving the Realism of Phage–Bacteria Chemostat Modeling

In this appendix, I note the key modifications that I made [84] to the Bohannon and Lenski [36] model toward greater predictive power, both for the sake of improved future modeling realism and to indicate where these modifications could be substantive. A first key modification was to assume that the Bohannon and Lenski chemostat was initiated with stationary phase rather than using log phase bacteria, thus requiring bacteria to go through an initial lag phase prior to their start of exponential growth. This is unlikely to impact the approaches presented here (Section 2.6), unless other chemostats that have been initiated with stationary phase bacteria are being modeled.

The second key modification was to assume that the robustness of both phage and bacterial population growth, when occurring, was less than had been determined initially, outside of chemostats. This is highly relevant to the approaches presented here to the extent that laboratory-determined phage growth parameters—adsorption rates, latent period, and burst size—might be optimistic relative to real-world circumstances. In other words, if you are predicting, using the various calculations presented here, that your treatments may just barely work in terms of sufficiently removing targeted bacteria, then in actuality, those treatments simply may not work. It is always important with phages, however, to recognize that their ability to replicate in situ can cover up what would otherwise be dosing insufficiencies, though this is without guarantee.

A last point has to do instead with the robustness of phage–virion survival. Based on the analysis of a number of published phage-containing chemostats beyond just that of Bohannon and Lenski [36] I found in many cases that rates of free phage decay could be greater than rates of chemostat outflow would suggest. In other words, something otherwise unaccounted for appeared to be either inactivating or removing phage virions in or from these various chemostats. I thus included a bacterium-independent free-phage decay parameter not only in the chemostat modeling found in Abedon [84] but also in the batch-culture phage–bacteria population dynamics model described in Equation (23). Similarly, and quite relevant to phage therapy, in many cases phage titers may decline in situ faster than simply free phage adsorption to bacteria would suggest. This likely would be beyond just considerations of the familiar (to phage therapists) expected declines in phage numbers over time during circulation in blood [110,111].
